# Comparative genomic analysis of *Enterococcus faecalis*: insights into their environmental adaptations

**DOI:** 10.1186/s12864-018-4887-3

**Published:** 2018-07-11

**Authors:** Qiuwen He, Qiangchuan Hou, Yanjie Wang, Jing Li, Weicheng Li, Lai-Yu Kwok, Zhihong Sun, Heping Zhang, Zhi Zhong

**Affiliations:** 1 Key Laboratory of Dairy Biotechnology and Engineering, Ministry of Education P.R. C, Inner Mongolia Agricultural University, Huhhot, 010018 People’s Republic of China; 2 Key Laboratory of Dairy Products Processing, Ministry of Agriculture P.R.C, Inner Mongolia Agricultural University, Huhhot, 010018 People’s Republic of China

**Keywords:** *Enterococcus faecalis*, Genome, Environment, Antibiotic resistance gene, Phylogeny

## Abstract

**Background:**

*Enterococcus faecalis* is widely studied as a common gut commensal and a nosocomial pathogen. In fact, *Enterococcus faecalis* is ubiquitous in nature, and it has been isolated from various niches, including the gastrointestinal tract, faeces, blood, urine, water, and fermented foods (such as dairy products). In order to elucidate the role of habitat in shaping the genome of *Enterococcus faecalis*, we performed a comparative genomic analysis of 78 strains of various origins.

**Results:**

Although no correlation was found between the strain isolation habitat and the phylogeny of *Enterococcus faecalis* from our whole genome-based phylogenetic analysis, our results revealed some environment-associated features in the analysed *Enterococcus faecalis* genomes. Significant differences were found in the genome size and the number of predicted open reading frames (ORFs) between strains originated from different environments. In general, strains from water sources had the smallest genome size and the least number of predicted ORFs. We also identified 293 environment-specific genes, some of which might link to the adaptive strategies for survival in particular environments. In addition, the number of antibiotic resistance genes was significantly different between strains isolated from dairy products, water, and blood. Strains isolated from blood had the largest number of antibiotic resistance genes.

**Conclusion:**

These findings improve our understanding of the role of habitat in shaping the genomes of *Enterococcus faecalis*.

**Electronic supplementary material:**

The online version of this article (10.1186/s12864-018-4887-3) contains supplementary material, which is available to authorized users.

## Background

*Enterococcus faecalis* (*E. faecalis*) is a Gram-positive coccoid bacterium occurring singly, in pairs, in short chains, or in groups [1]; and it is the most common species within the genus *Enterococcus*. Many *E. faecalis* strains are associated with infections, including urinary tract infection, bacteraemia, endocarditis, neonatal infection, and infection of the central nervous system [2, 3]. Some *E. faecalis* strains have developed resistance to several antibiotics including vancomycin, which is the last line of defence against a wide range of multi-resistant Gram-positive pathogens [4]. The first vancomycin-resistant clinical strain of *E. faecalis* was reported in 1989 in the United States [5]. At present, *E. faecalis* is emerging as an important cause of hospital acquired infection and multidrug resistance [6]. For these reasons, *E. faecalis* is not generally regarded as safe (GRAS) [7].

*Enterococcus faecalis* is ubiquitous in nature and has been isolated from many different niches. The gastrointestinal (GI) tract of humans and animals is commonly considered as the primary habitat of *E. faecalis*, where it occurs as a commensal [6, 8, 9]. In addition, the blood and urine specimens of humans and animals are also major sources of *E. faecalis* [6]. As a species of lactic acid bacteria, *E. faecalis* is widely used in the production of fermented foods, particularly fermented dairy products. In recent years, numerous *E. faecalis* strains have been recovered from traditional dairy products [10–13]. Furthermore, water and soil are also common habitats of *E. faecalis* [14–16]. Considering the primary habitat of *E. faecalis* as the GI tract but meanwhile its wide distribution across numerous other niches makes it interesting to understand the relationship between enteric and extra-enteric *E. faecalis* strains. A key question is whether the extra-enteric *E. faecalis* strains are a product of faecal pollution or if they exist as independent lineages. Another intriguing aspect is how *E. faecalis* survives in and adapts to the highly diverse environments. Nowadays microbial genome sequencing provides an opportunity to answer these questions.

The complete genome of *E. faecalis* V583, one of the first reported vancomycin-resistant strains, was published in 2003 [17]. Subsequently, the genome sequences of 28 enterococcal strains (including 18 *E. faecalis* strains) were comparatively analysed to identify distinctive genetic traits and biochemical functions between lineages of clinical and environmental importance [18]. In 2014, the genomes of 38 *E. faecalis* strains were analysed to distinguish clinical from nonclinical strains [19]. In 2016, Raven et al. sequenced the whole genomes of three epidemic lineages isolated in the UK, analysed the genome-level data of altogether 168 *E. faecalis* strains, and described the evolution of vancomycin resistance within this strain collection [20]. As more and more genomes of *E. faecalis* from different habitats are available, we are now in a better position to understand the molecular basis of their environmental adaptation using comparative genomic analysis.

In this study, a total of 78 genomes of *E. faecalis* (including 15 genomes sequenced in this work and 63 genomes retrieved from the Genbank database) were subjected to comparative genomic analysis. These strains were isolated from faeces, blood, urine, dairy products, and water. We believe that results from this comparative genomic analysis can provide the insight necessary to understand the genetic relationships between these *E. faecalis* strains and the adaptive mechanisms that have evolved to allow them to occupy different niches.

## Methods

### Bacterial strains

A total of 78 genomes of *E. faecalis* were subjected to comparative genomic analysis. Among the 78 strains, 15 strains were collected from China, Russia, and Mongolia by our laboratory. These strains were isolated from naturally fermented dairy products and their genomes were sequenced in this study (Additional file 1). The other 63 genomes of *E. faecalis* were retrieved from the Genbank database (Additional file 1). To ensure meaningful comparison between genomes and to analyse how the isolation habitat affected the genome characteristics, more than 400 genome sequence records of *E. faecalis* in the Genbank database were scanned. Only 63 genomes (including five complete genomes) were selected for this study based on a clear documentation of the strain isolation source and a high sequencing quality. The 63 strains were isolated from blood (20 strains), faeces (16 strains), urine (10 strains), dairy products (3 strains), water (11 strains), oral cavity (1 strain), and multiple sites (2 strains) (Additional file 1).

### DNA extraction

Strains were cultured under anaerobic conditions in Man Rogosa and Sharpe (MRS) broth at 37 °C. DNA was extracted from each strain using a bacterial DNA extraction kit (OMEGA D3350–02) according to the manufacturer’s instructions. Genomic DNA was quantified using a TBS-380 fluorometer (Turner BioSystems Inc., Sunnyvale, CA). Only high-quality DNA samples (OD260/280 = 1.8~ 2.0, > 6 μg) were used to construct fragment libraries (200 to 300 bp).

### Sequencing, assembly, coding sequence (CDS) prediction, and annotation

The whole-genome sequencing was done using the Illumina MiSeq platform (Illumina Inc., U.S.A) by generating 2 × 150 bp paired-end libraries using the Nextera DNA Sample Preparation Kit (Illumina Inc., U.S.A) following the manufacturer’s instructions. On average, 625 Mb of high-quality data were generated for each strain, corresponding to 176- to 247-fold sequencing depth (Additional file 1).

The paired-end reads were first assembled de novo using SOAPdenovo v1.06 [21]. Local inner gaps were filled and single base errors were corrected using the software GapCloser (http://sourceforge.net/projects/soapdenovo2/files/GapCloser/). Coding sequences were predicted for each sequenced genome using Glimmer v3.02 [22]. Functional annotation of predicted open reading frames (ORFs) was achieved using RAST 2.0 [23] and COG database [24]. The individual genome assemblies of the 15 strains generated in this work were deposited in the National Center for Biotechnology Information under the accession numbers of MSQG00000000 to MSQU00000000 (Additional file 1).

### Construction of core- and pan-genomes

The core- and pan-genomes of *E. faecalis* were constructed based on the families of homologous genes. The families of homologous genes for *E. faecalis* were computed using the SiLiX software [25]. Briefly, a pair of ORFs would be classified into the same gene family when their amino acid sequence identity value was above 80% and when the amino acid sequence alignment length spanned more than 80% of the longer ORF. All predicted ORFs of the 78 genomes were firstly grouped into their respective gene families before pan-genome construction. The pan-genome was constructed by counting the total number of non-redundant gene families within the complete dataset. The core-genome was constructed by counting the total number of gene families commonly shared by all genomes. The sequence of the longest ORF from each gene family was selected as the representative gene for functional annotation and phylogenetic reconstruction.

### Phylogenetic analysis

A phylogenetic tree was constructed using the core genes of the 78 strains of *E. faecalis*. We first aligned the nucleotide sequences of the core genes using MUSCLE v3.8.31 [26], followed by removing the unreliable alignment regions and intragenic homologous recombination using Gblocks (http://molevol.cmima.csic.es/castresana/Gblocks.html) and Gubbins (http://www.sanger.ac.uk/science/tools/gubbins), respectively. A maximum likelihood tree was constructed based on the concatenated alignments using FastTree 2.1.8 [27] with 10,000 bootstrap iterations.

### Identification of environment-specific genes

The subset of variable genes in the pan-genome was analysed to determine whether their distribution was significantly associated with the strain isolation niche (dairy, blood, faeces, urine, water, and oral cavity). If the frequency of a gene present in strains from one niche was much higher than the overall occurrence across all 78 strains, this gene was considered environment-specific. Scoary 1.6.16 (run with 1000 permutation replicates) was used to identify the spectrum of environment-specific genes, and the results were corrected for multiple testing. A *p*-value of less than 0.05 after Benjamini-Hochberg correction was considered significant [28].

### Identification of antibiotic resistance genes

A BLAST search was performed with all predicted ORFs from the 78 strains against the Comprehensive Antibiotic Resistance Database (CARD; http://arpcard.mcmaster.ca) to identify potential antibiotic resistance genes (E-value of <1e-15 and sequence identity > 85%) [29].

### Identification of virulence factors

A BLAST search was performed with all predicted ORFs from the 78 strains against the Virulence Factor Database (VFDB) to identify genes encoding known virulence factors (E-value <1e-15 and sequence identity > 95%) [30].

### Prophage identification

Intact and incomplete prophage regions were identified through an integrated search with the annotation tool, PHASTER [31]. This involved genome-scale ORFs prediction and translation (via GLIMMER), protein identification (via BLAST matching and annotation by homology), phage sequence identification (via BLAST matching to a phage-specific sequence database), tRNA identification, and attachment site recognition. Only intact regions were analysed in-depth.

### Statistical analysis

Data are presented as means ± SEM. One-way ANOVA followed by Tukey’s post-hoc test was used for statistical significance determination using SPSS Statistics 19 (IBM, Armonk, New York, USA). Significance was set at *p*-value < 0.05.

## Results

### General genomic characteristics of the species *Enterococcus faecalis*

**Fig. 1:**
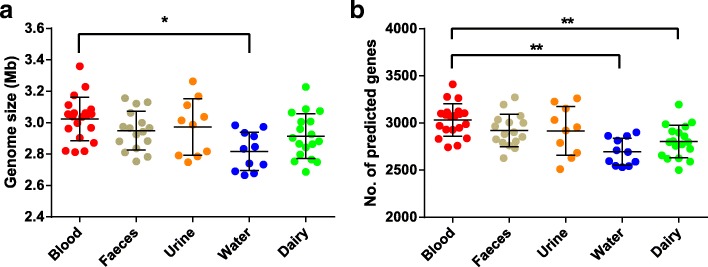
Genome size (**a**) and number of predicted open reading frames (ORFs) (**b**) of *Enterococcus faecalis* strains isolated from different niches. An asterisk (*) indicates a *p*-value < 0.05; double asterisks (**) indicate a *p*-value < 0.01 (one-way ANOVA test)

The analysed *E. faecalis* genomes had a low G + C content ranging from 37.0 to 38.0% (Additional file 1). The average genome size was 2.94 ± 0.15 Mb, with 2884 ± 211 predicted ORFs (Additional file 1). Furthermore, there were significant differences in the genome size and the number of predicted ORFs between strains isolated from different sources (Fig. 1). On average, strains isolated from water sources had the smallest genome size and the lowest number of predicted ORFs, which were significantly different from those originated from blood (*P* < 0.01). In addition, strains from dairy products and blood differed significantly in their number of predicted ORFs (*p* < 0.01). There were no significant differences in the genome size nor the number of predicted ORFs between strains isolated from blood, faeces, and urine samples.

### The pan- and core-genome of the species *Enterococcus faecalis*

**Fig. 2:**
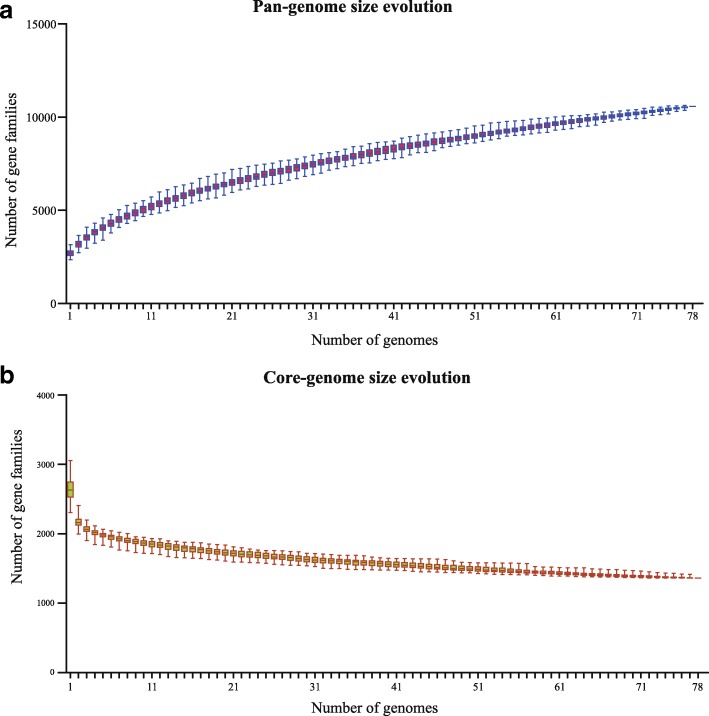
Accumulation curves for pan-genomes (**a**) and core-genomes (**b**) of the species *Enterococcus faecalis.* The procedure was repeated 1000 times by randomly modifying the order of integration of genomes

The pan-genome of the 78 *E*. *faecalis* strains was composed of 10,573 gene families; and the pan-genome size grew continuously with the increase in newly deciphered genomes (Fig. 2a). In contrast, the size of the core-genome gradually stabilized and the increase in genome number had little influence on the core-genome size when the number of genomes reached 60–70 (Fig. 2b). The core gene set comprised 1361 genes, i.e. 47.2% of the number of predicted ORFs (2884 per genome), suggesting that over half of the predicted ORFs in each genome were dispensable.

**Fig. 3:**
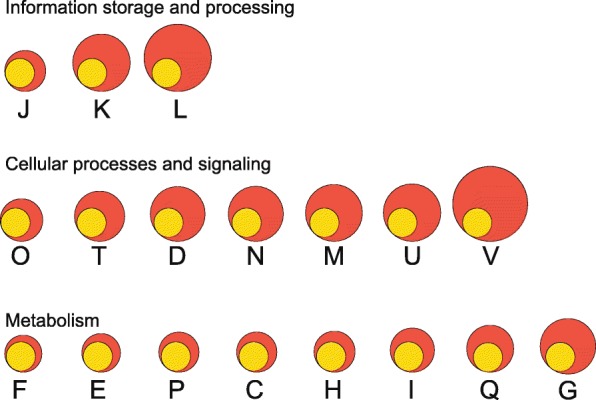
Expansion of the pan-genome compared with the core-genome for each functional category. The number of core genes in each functional category was normalized (yellow circle). The magnification of the pan genes compared with the core genes in each functional category is shown by the red circle. The letter below the circle represents the functional category: [J] Translation, ribosomal structure and biogenesis; [K] Transcription; [L] Replication, recombination and repair; [O] Posttranslational modification, protein turnover, chaperones; [T] Signal transduction mechanisms; [D] Cell cycle control, cell division, chromosome partitioning; [N] Cell motility; [M] Cell wall/membrane/envelope biogenesis; [U] Intracellular trafficking, secretion, and vesicular transport; [V] Defence mechanisms; [F] Nucleotide transport and metabolism; [E] Amino acid transport and metabolism; [P] Inorganic ion transport and metabolism; [C] Energy production and conversion; [H] Coenzyme transport and metabolism; [I] Lipid transport and metabolism; [Q] Secondary metabolites biosynthesis, transport and catabolism; [G] Carbohydrate transport and metabolism

Functional analysis of the representative genes in the pan- and core-genome was conducted using the COG database (Table 1). The core genes were mainly distributed in four categories, representing amino acid transport and metabolism; transcription; translation, ribosomal structure and biogenesis; and carbohydrate transport and metabolism. These genes together accounted for 33.4% of the core-genome. As a large proportion of genes were dispensable, pan-genome expansion happened in each functional category in varying degree compared with the core-genome (Fig. 3). The largest extent of expansion occurred in the group of genes involved in defence mechanisms (COG category [V]). Only 27 out of the 181 defence mechanisms-related genes within the pan-genome were core genes. The smallest extent of expansion occurred in the COG categories of nucleotide transport and metabolism [F] and amino acid transport and metabolism [E]. More than half of the pan genes in these two functional categories were also core genes. These results suggest that *E*. *faecalis* possesses multiple defence mechanisms, while genes involved in nucleotide and amino acid transport and metabolism are generally more conserved.

**Table 1:** Functional categories of core and pan genes in 78 *Enterococcus faecalis* genomes

COG Functional category	No. of core genes	No. of pan genes	Proportion of core genes among pan genes (%)
Translation, ribosomal structure and biogenesis	111	221	50.2
RNA processing and modification	0	0	0.0
Transcription	114	448	25.4
Replication, recombination and repair	70	378	18.5
Chromatin structure and dynamics	0	0	0.0
Cell cycle control, cell division, chromosome partitioning	14	50	28.0
Nuclear structure	0	0	0.0
Defense mechanisms	27	181	14.9
Signal transduction mechanisms	52	156	33.3
Cell wall/membrane/envelope biogenesis	54	208	26.0
Cell motility	5	18	27.8
Cytoskeleton	0	1	0.0
Extracellular structures	0	1	0.0
Intracellular trafficking, secretion, and vesicular transport	15	59	25.4
Posttranslational modification, protein turnover, chaperones	39	84	46.4
Energy production and conversion	74	142	52.1
Carbohydrate transport and metabolism	107	410	26.1
Amino acid transport and metabolism	123	217	56.7
Nucleotide transport and metabolism	61	99	61.6
Coenzyme transport and metabolism	44	88	50.0
Lipid transport and metabolism	32	74	43.2
Inorganic ion transport and metabolism	86	164	52.4
Secondary metabolites biosynthesis, transport and catabolism	19	50	38.0
General function prediction only	172	439	39.2
Function unknown	133	560	23.8

### Phylogeny of the species *Enterococcus faecalis*

**Fig. 4:**
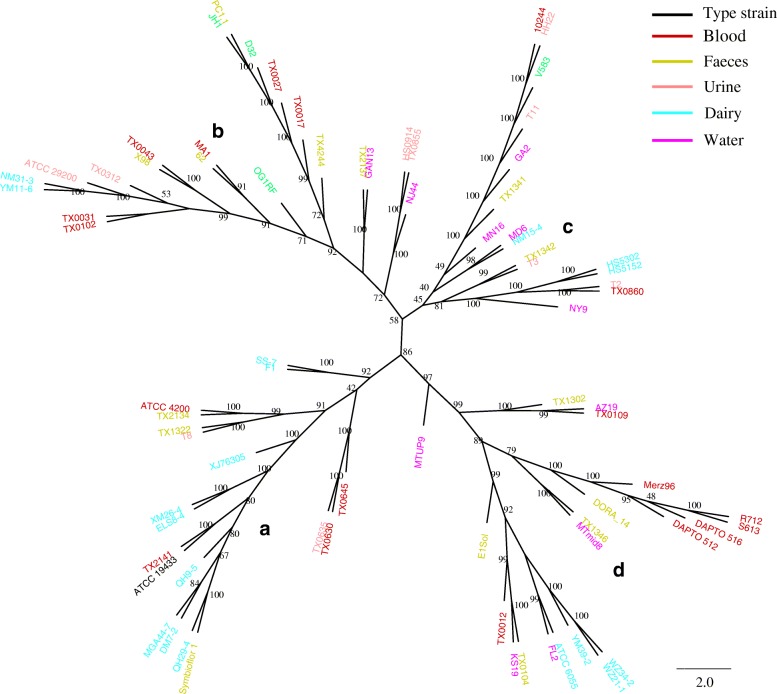
Phylogenetic tree constructed based on the core genes of 78 *Enterococcus faecalis* strains

To investigate the phylogenetic relationship between the 78 strains, the concatenated nucleotide sequence of the core genes of each strain was used to construct a phylogenetic tree. The 78 strains were divided into four branches on the phylogenetic tree (Fig. 4). The type strain (ATCC 19433) fell under branch A, which contained 19 strains, including seven dairy strains. Branch B comprised 22 strains. Branch C was the smallest branch with only 16 strains. Branch D composed of 21 strains of which none was isolated from urine samples. Strains originated from different niches were evenly dispersed across the four branches, suggesting no correlation between strain isolation niche and strain phylogeny.

### Environment-specific genes

**Fig. 5:**
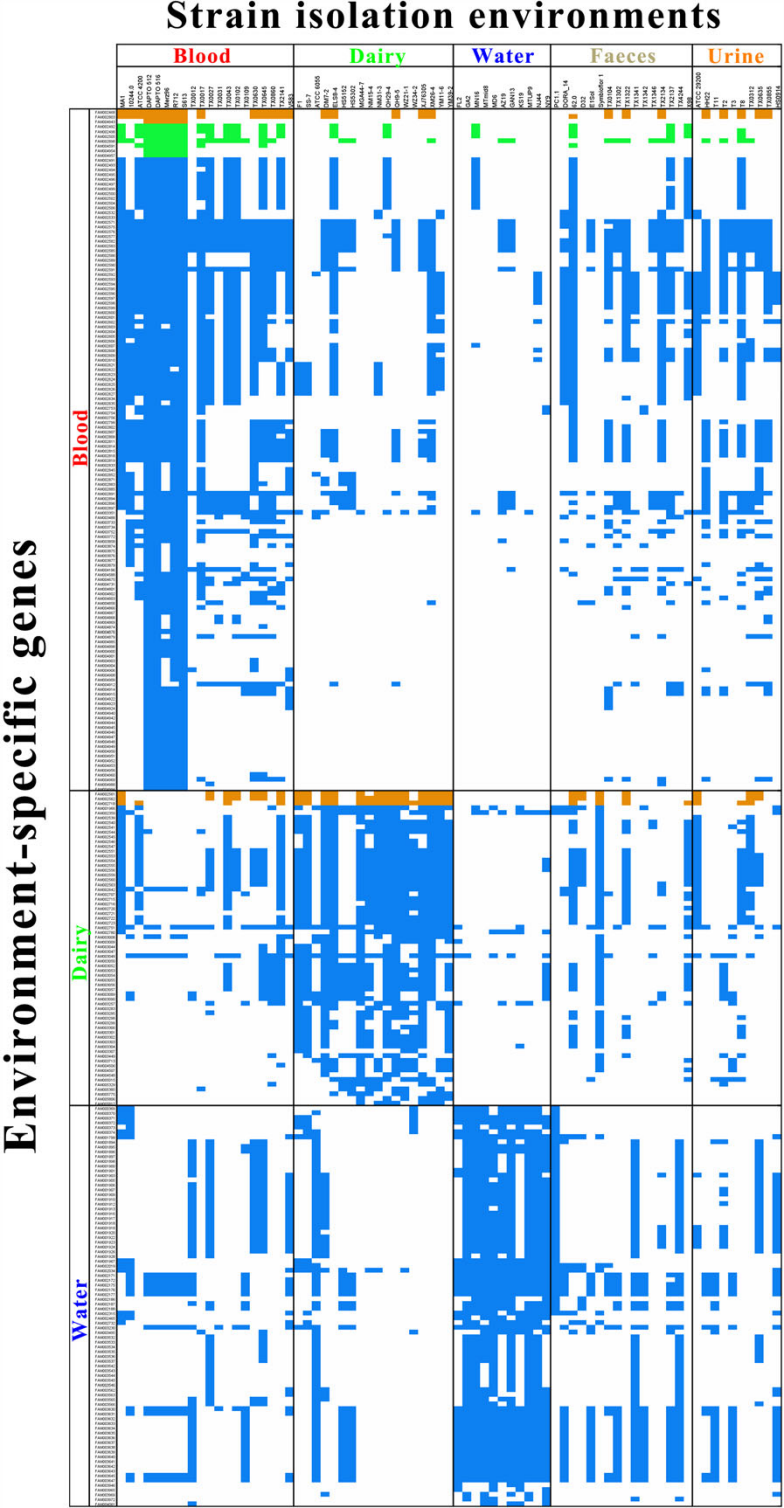
Heatmap of environment-specific genes. The blue dots represent genes present in specific strains. Genes annotated as “transposase” and “phage protein” are highlighted in yellow and green, respectively

Pan-GWAS analysis was performed with Scoary to identify genes that were present in strains associated with a particular environment [28]. A total of 293 environment-specific genes were identified, including 143, 66, and 84 genes that were specifically linked to strains isolated from blood, dairy, and water sources, respectively (Fig. 5). Most of the environment-specific genes encoded hypothetical proteins with unknown function. The environment-specific genes of known function were analysed using the COG database; and most of them were involved in carbohydrate transport and metabolism (Additional file 2).

Among the blood-specific genes, five genes were involved in galactose metabolism: dgoD encodes a galactonate dehydratase; PTS-Aga-EIID encodes the N-acetylgalactosamine-specific IID component of phosphotransferase system (PTS); PTS-Gat-EIIA, B, and C encode the galactitol-specific IIA, B, and C components of PTS system, respectively. Furthermore, panD encodes an aspartate 1-decarboxylase that involves in beta-alanine metabolism; eda encodes the 2-dehydro-3-deoxyphosphogluconate aldolase / (4S)-4-hydroxy-2-oxoglutarate that involves in carbon metabolism; znuA encodes a zinc transport system substrate-binding protein.

Among the dairy-specific genes, the genes phnC, phnD, and phnE encode for the phosphonate transport system ATP-binding protein, phosphonate transport system substrate-binding protein, and phosphonate transport system permease protein, respectively. Three genes constitute the ABC transporter of phosphonate. PTS-Cel-EIIC and PTS-Lac-EIIC encode the cellobiose-specific IIC component and lactose-specific IIC component of the PTS system, respectively. PldB encodes a lysophospholipase that involves in glycerophospholipid metabolism. Dld encodes a D-lactate dehydrogenase that involves in pyruvate metabolism. ClpL encodes an ATP-dependent Clp protease ATP-binding subunit.

Among the 84 water-specific genes, 28 were present in all 11 strains isolated from water sources. Among these, cbiO encodes an ATP-binding protein, while cbiM and cbiQ both encode permease proteins. Three genes were involved in the cobalt and nickel transport system: znuC is a zinc transport system ATP-binding protein involved in zinc transport; the genes PTS-Man-EIIB and PTS-Man-EIIC encode mannose-specific IIB and IIC components of PTS system, respectively. DdhP encodes an alcohol dehydrogenase involved in tyrosine metabolism and fatty acid degradation. PcaC encodes a 4-carboxymuconolactone decarboxylase involved in benzoate degradation. RP-L33 encodes the large subunit ribosomal protein L33.

### Antibiotic resistance genes

Potential antibiotic resistance genes were detected by blasting the 78 *E. faecalis* genomes against the CARD database (Additional file 3). There were on average 7.5 antibiotic resistance genes in each genome. The number of antibiotic resistance genes varied greatly between strains. Four blood originated strains (DAPTO 516, DAPTO 512, S613, and R712) and one faecal strain (TX0104) had the highest number of antibiotic resistance genes; each of them possessed 18 antibiotic resistance genes. Thirty-four strains contained the fewest antibiotic resistance genes, with only five antibiotic resistance genes per genome.

**Fig. 6:**
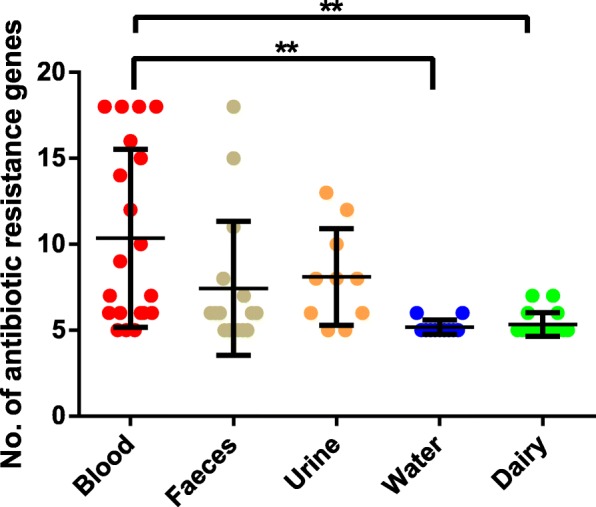
The number of antibiotic resistance genes in strains isolated from different niches. Double asterisks (**) indicate a *p*-value < 0.01 (one-way ANOVA test)

Based on the average number of antibiotic resistance genes, the 78 investigated strains could be divided into three classes. The first class included strains isolated from blood with the largest number of antibiotic resistance genes (10.4 antibiotic resistance genes per strain). The second class included strains isolated from faeces (7.4 antibiotic resistance genes per strain) and urine (8.1 antibiotic resistance genes per strain). The last class included strains from dairy (5.3 antibiotic resistance genes per strain) and water sources (5.2 antibiotic resistance genes per strain) with the fewest antibiotic resistance genes. Furthermore, the number of antibiotic resistance genes present in the blood originated strains was significantly different from those isolated from dairy and water sources (*p* < 0.01) (Fig. 6).

Although the distribution of antibiotic resistance genes varied greatly between strains, five antibiotic resistance genes were commonly present in all 78 strains. Most of these antibiotic resistance genes were involved in efflux-mediated resistance to antibiotics. LsaA encodes an ABC efflux pump. It confers resistance to clindamycin, quinupristin-dalfopristin, and dalfopristin. EmeA encodes a multidrug efflux pump [32]. EfrA and efrB encode two subunits of the EfrAB efflux pump, which are related to the drug resistance in both *E. faecalis* and *E. faecium*. In addition to the efflux-mediated resistance genes, dfrE encodes a dihydrofolate reductase that confers resistance to trimethoprim.

Moreover, we found two types of vancomycin-resistance gene clusters among the 78 strains: vanA-type and vanB-type. The vanA-type cluster is 8.1 kb, containing six vancomycin-resistant genes: vanZA, vanYA, vanXA, vanHA, vanSA, and vanRA [33]. The vanA-type cluster was only found in four strains isolated from blood (DAPTO 512, DAPTO 516, R712, and S613). The vanB-type is 6.4 kb, containing six vancomycin-resistant genes: vanRB, vanSB, vanYB, vanWB, vanHB, and vanXB [33]. The vanB-type cluster was exclusively identified in two strains of blood origin (V583 and Merz96) and one faecal strain (TX0104).

### Virulence factors

Genes coding for known virulence factors were identified by blasting the 78 *E. faecalis* genomes against the VFDB database. Sixty putative virulence factors (23.8 per genome) were detected within the 78 *E. faecalis* genomes (Additional file 4). The number of virulence factors varied greatly between strains. The blood originated strain, V583, had the highest number of virulence factors (52 within the genome), contrasting to the strains E1Sol, TX1322 (both isolated from faeces samples), and T3 (isolated from a urine specimen) that carried only a low number of nine virulence factors per strain. Furthermore, no significant difference existed in the number and functions of virulence factors between strains of different isolation sources.

In summary, the virulence factors of *E. faecalis* were mainly involved in adherence, antiphagocytosis, biofilm formation, quorum sensing system, production of exoenzymes and toxins. The most common virulence factors were responsible for adherence (24 out of the 60 putative virulence factors), including ebpA/B/C (encode three Ebp pili subunits that facilitate bacterial adherence to host extracellular matrix proteins), ace (encodes a collagen adhesin), and asa1 (encodes aggregation substance). Another common putative virulence factor present in *E. faecalis* was the csp operon. The csp operon consists of 11 ORFs (i.e. cpsA to cpsK), encoding an antiphagocytosis factor that facilitates bacterial evasion of the host immune system. Moreover, some members of the cytolysin (cyl) operon were detected in the *E. faecalis* genomes. Normally, the cyl operon comprises eight genes, i.e. cylA/B/I/L/M/R1/R2/S. However, only four genes, cylA/B/I/M, were detected in five of the studied strains, including two urine isolated strains (T2 and T8), two dairy strains (SS-7 and F1), and one blood originated strain (Merz96).

### Prophage sequences

A total of 116 intact prophages were identified (Additional file 5). Not all investigated *E. faecalis* genomes contained intact prophages (detected only in 65 out of 78 genomes). No apparent correlation was found between the isolation source and occurrence of intact prophage sequences. The 13 strains that contained no intact prophage were originated from all types of environments, including four dairy strains (HS5152, HS5302, WZ34–2, XJ76305), three water originated strains (GA2, KS19, MTmid8), two faecal strains (TX0104, TX4244), two urine isolated strains (HH22, T11), one blood isolated strain (TX0031), and one oral strain (OG1RF).

The strain TX0645 (isolated from blood), ATCC6055 (isolated from dairy), and X98 (isolated from faeces) contained the highest number of intact prophages (four per genome). Apart from some common enterococcal prophages (e.g. phiEf11, phiFL4A, SANTOR1, and vB), prophages associated with other bacterial genera (including *Lactobacillu*s, *Lactococcus*, *Listeria*, *Bacillus*, *Clostridium*, *Staphylococcus*, *Streptococcus*, and *Weissella*) were also detected. Such results together suggest that prophages are extensively propagated intra- and inter-species; and their spread is independent from the isolation source.

## Discussion

In this study, we performed a comparative genomic analysis of 78 *E. faecalis* strains (15 genomes sequenced by this work plus 63 genome sequences retrieved from the Genbank database). These strains were originated from a wide range of sources, including blood, faeces, urine, dairy products, water, and oral cavity. Our work has taken the advantage of the wide ecological niches of these strains to elucidate the role of habitat in shaping the genome characteristics of the *E*. *faecalis* species.

The pan-genome of the 78 *E*. *faecalis* strains comprised 10,573 gene families, of which 1361 genes were conserved across all studied strains. This corroborates the results derived from the previous analysis of 168 strains [20]. Comparing with the core-genome, the biggest pan-genome expansion occurred in the subset of defence mechanisms-related genes. Only 14.9% (27 out of 181) of the defence mechanisms-related genes were core genes. The 27 core genes mainly encode the ATP-binding cassette (ABC) transport system permease and ABC subfamily B, which confer multi-drug resistance to the bacteria [34]. The variable gene portion mainly encodes restriction endonucleases of type I restriction modification system, which function to defend against invading viruses, such as bacteriophage [35, 36]. The type I restriction modification system consists of the R, M, and S subunits. Our study identified mainly the S subunit that is responsible for determining the specificity of the DNA-binding site recognition during DNA cleavage and modification of the enterococcal genomes [37, 38]. The high variability of this spectrum of genes suggests the existence of multiple defence mechanisms to protect the cells from viral invasion. Furthermore, the defence mechanisms of *E. faecalis* do not seem to be associated with the strain isolation habitat, as no environment-specific genes were identified within this functional category.

We then performed a phylogenetic reconstruction using the core-genome of all 78 *E*. *faecalis* strains. The results suggested no correlation between the strain isolation habitat and phylogeny, which corroborates the inference drawn by a previous study [39]. However, our in-depth analysis of the functional genomes did reveal environment-specific adaptation in *E. faecium* strains originated from dairy products, water, and blood.

Milk is a lactose-rich environment. Some of the identified dairy-specific genes seem to help the dairy enterococci utilize the lactose present in the milk environment. For example, the dairy-specific gene dld encodes D-lactate dehydrogenase, which has previously been found in plasmids from *Lactococcus lactis* and *Lactobacillus delbrueckii subsp. bulgaricus*. D-lactate dehydrogenase may involve in D-lactate utilization under aerobic conditions [40]. During milk fermentation, D-lactate utilization might result in pH reduction or the sugar could be converted to acetate accompanied with ATP production; both activities would enhance bacterial survival in dairy products. Siezen et al. suggested that the dld gene might have been acquired from Gram-negative bacteria by horizontal transfer, as the best homologues (about 50% sequence identity and the same size) are found exclusively in Gram-negative bacteria such as *Escherichia coli* and *Shigella* spp. but not in any Gram-positive bacteria [40].

Another set of dairy-specific genes was lacE, lacF, and lacG. In Gram-positive bacteria, lactose is internalized by the phosphoenolpyruvate-dependent PTS, which consists of the lactose-specific IIBC and IIA components (encoded by lacE and lacF, respectively), yielding lactose-6-phosphate. Lactose-6-phosphate is then hydrolyzed to glucose and galactose-6-phosphate by a cytoplasmic phospho-β-galactosidase (encoded by lacG) [41].

Among the water-specific genes, cbiM, cbiO, and cbiQ encode part of the cobalt and nickel transport system. Furthermore, metal uptake operons were widely distributed in all 11 strains from water sources. This result is in line with the study of [9]. The transition metals, nickel and cobalt, are essential cofactors for many prokaryotic enzymes involved in a variety of metabolic processes [42]. The mean concentration of nickel in freshwater environments is about 10 μg/L, which is much lower than other environments [14]. Thus, a high-affinity nickel uptake system may be beneficial to the survival of the water-dwelling *E. faecalis* strains.

Interestingly, some environment-specific genes do not seem to have any adaptive relationship with the isolation habitat. For example, the dairy-specific genes phnC, phnD, and phnE constitute the integrated ABC transporter of phosphonates, which are quite common among many organisms, ranging from bacteria, fungi, molluscs, insects, plants, and animals. These three genes were identified in 15 of the 18 dairy-associated *E. faecalis* strains. However, there is no indication as to why the integrated ABC transporter of phosphonates should be specifically enriched in dairy strains. Nevertheless, the precise role of natural phosphonates is still poorly understood. A similar situation occurred with the blood-specific genes PTS-Gat-EIIA, PTS-Gat-EIIB, and PTS-Gat-EIIC, which constitute the PTS system involved in galactitol metabolism. To our knowledge, plasma galactitol is only an important parameter for the assessment of steady-state galactose metabolism in galactosaemia [43, 44]. A large number of environment-specific genes are hypothetical genes with unknown function. These genes may encode additional environment-associated functions and require further research.

Another interesting phenomenon observed in this work was the differences in the antibiotic resistance gene profile between strains isolated from various niches. Strains from blood sources had significantly more antibiotic resistance genes than those from dairy and water sources. This is in line with the results of Raven et al. that reported an enrichment of antibiotic resistance genes in epidemic lineages [20]. The pressure of natural selection may play a key role in the uneven distribution of antibiotic resistance genes in this case. For example, the tetM gene codes a ribosomal protection protein that confers tetracycline resistance. This gene is known to associate with or even encoded by transposable DNA elements, and its horizontal transfer between bacterial species has been documented [45]. Our study showed that tetM was present mostly in strains isolated from blood (15 out of 20) and occurred in much lower frequency in strains originated from dairy (4 out of 18) and water (2 out of 11) sources. Tetracyclines have a broad spectrum of antibiotic action and are commonly used in treating bacterial infections; thus, strains from blood sources are more likely to be exposed to the bacteriostatic activity of tetracyclines and that strains lacking tetracycline resistance genes would be outcompeted rapidly. This explains the high proportion of tetM-positive blood originated strains. In contrast, strains from dairy and water sources would not need tetracycline resistance genes for survival because of the much lower environmental selection pressure of tetracycline; thus, these genes were not maintained in the bacterial genomes.

Five antibiotic resistance genes were found among the 78 strains, although the number of antibiotic resistance genes found in each strain varied greatly. Most of these antibiotic resistance genes were involved in efflux-mediated resistance to antibiotics. Efflux was first described as a mechanism of resistance to tetracycline. In recent years, numerous plasmid- and chromosome-encoded efflux mechanisms have been described in various microorganisms [46]. The fact that the efflux-mediated resistance is coded by core genes implicates that it is an important intrinsic resistance mechanism of *E. faecalis*.

Previous studies have characterised six types of vancomycin resistance in enterococci [33]. Our study found two types of vancomycin-resistant gene clusters among the 78 strains, i.e. vanA and vanB type clusters. VanA is the most frequently encountered type of glycopeptide resistance in enterococci; strains that have acquired vanA are resistant to high levels of vancomycin [33]. Raven et al. found that nearly all vancomycin-resistant *E. faecalis* carried vanA cluster [20]. Our study identified the vanA type gene cluster only in four blood originated strains. It is interesting to note that these four strains were most closely related genetically (Fig. 4). This may indicate that the common ancestor of these four strains had acquired the vanA type resistant gene cluster before being internalized in this lineage. The organization and functionality of the vanB type cluster is similar to that of vanA, but the resistant levels are variable [33]. Among the isolates we studied, the three vanB-positive strains were isolated from blood and faeces. Our results together show that the blood isolated strains had more vancomycin-resistant genes than those from other sources.

There are some limitations of this work. The strain collection used was relatively small for genome-wide study, which might lead to bias of some results, such as the identified environment-specific genes. Moreover, in addition to isolation habitat, other factors, e.g. geographic origin and year of collection, might also involve in shaping the bacterial genomes. However, due to incomplete strain documentation in some of the retrieved records, our work could not cover these aspects.

## Conclusions

In summary, although there was no correlation between the strain isolation source and phylogeny, our results did demonstrate that habitat was involved in shaping the *E. faecalis* genomes. There were significant differences in the genome size and number of predicted ORFs between strains isolated from different habitats. Furthermore, some environment-specific genes were found in strains isolated from dairy, blood, and water sources; and some of these genes might improve the adaptive capacity of the strains to survive in their dwelling environment. In addition, strains from blood had the largest number of antibiotic resistance genes. All these findings suggest that the natural habitat where the strain was recovered is effective in shaping the *E. faecalis* genomes.

## Additional files

Additional file 1: List of the *Enterococcus faecalis* strains analyzed in this study. The strain and genome information is listed in this table. (XLS 17 kb)

Additional file 2: List of environment-specific genes in the investigated *Enterococcus faecalis* strains. A total of 293 environment-specific genes were identified, including 143, 66, and 84 genes that were specifically linked to strains isolated from blood, dairy, and water sources, respectively. The function of environment-specific genes was annotated based on the COG, KEGG, and nr databases. (XLS 69 kb)

Additional file 3: Antibiotic resistance genes found in the 78 *Enterococcus faecalis* strains. Potential antibiotic resistance genes were detected by blasting the 78 *E. faecalis* genomes against the CARD database (E-value of <1e-15 and sequence identity > 85%). (XLS 94 kb)

Additional file 4: Virulence factors found in the 78 *Enterococcus faecalis* strains. Genes coding for known virulence factors were identified by blasting the 78 *E. faecalis* genomes against the VFDB database (E-value <1e-15 and sequence identity > 95%). Sixty putative virulence factors (23.8 per genome) were detected within the 78 *E. faecalis* genomes. (XLS 237 kb)

Additional file 5: Distribution of the intact prophage regions among the *Enterococcus faecalis* strains. Intact prophage regions were identified through PHASTER. A total of 116 intact prophages were identified. (XLS 45 kb)

## Abbreviations

ABC: ATP-binding cassette
CARD: Comprehensive Antibiotic Resistance Database
E. faecalis: Enterococcus faecalis
GI: Gastrointestinal
GRAS: Generally regarded as safe
VFDB: Virulence Factor Database
